# High-density spinal cord stimulation selectively activates lower urinary tract nerves

**DOI:** 10.1088/1741-2552/aca0c2

**Published:** 2022-11-22

**Authors:** Maria K Jantz, Chaitanya Gopinath, Ritesh Kumar, Celine Chin, Liane Wong, John I Ogren, Lee E Fisher, Bryan L McLaughlin, Robert A Gaunt

**Affiliations:** 1 Rehab Neural Engineering Labs, University of Pittsburgh, Pittsburgh, PA, United States of America; 2 Department of Bioengineering, University of Pittsburgh, Pittsburgh, PA, United States of America; 3 Center for the Neural Basis of Cognition, Pittsburgh, PA, United States of America; 4 Department of Physical Medicine and Rehabilitation, University of Pittsburgh, Pittsburgh, PA, United States of America; 5 Biomedical Engineering, Carnegie Mellon University, Pittsburgh, PA, United States of America; 6 Micro-Leads Inc., Somerville, MA, United States of America

**Keywords:** bladder, spinal cord stimulation, peripheral nervous system, autonomic nervous system, neuromodulation

## Abstract

*Objective.* Epidural spinal cord stimulation (SCS) is a potential intervention to improve limb and autonomic functions, with lumbar stimulation improving locomotion and thoracic stimulation regulating blood pressure. Here, we asked whether sacral SCS could be used to target the lower urinary tract (LUT) and used a high-density epidural electrode array to test whether individual electrodes could selectively recruit LUT nerves. *Approach*. We placed a high-density epidural SCS array on the dorsal surface of the sacral spinal cord and cauda equina of anesthetized cats and recorded the stimulation-evoked activity from nerve cuffs on the pelvic, pudendal and sciatic nerves. *Main results*. Here we show that sacral SCS evokes responses in nerves innervating the bladder and urethra and that these nerves can be activated selectively. Sacral SCS always recruited the pelvic and pudendal nerves and selectively recruited both of these nerves in all but one animal. Individual branches of the pudendal nerve were always recruited as well. Electrodes that selectively recruited specific peripheral nerves were spatially clustered on the arrays, suggesting anatomically organized sensory pathways. *Significance.* This selective recruitment demonstrates a mechanism to directly modulate bladder and urethral function through known reflex pathways, which could be used to restore bladder and urethral function after injury or disease.

## 1. Introduction

Lower urinary tract (LUT) dysfunction occurs in 20%–40% of the global population [1] and has an economic impact measured in billions of dollars in medical costs every year [2]. One common clinical problem is overactive bladder; people experience excessive bladder contractions that increase the frequency with which they feel the urge to void [3]. Overactive bladder reduces sleep quality, participation in daily activities, and is associated with increased incidence of urinary tract infections [3]. Furthermore, losing voluntary bladder control is one of the least visible but most limiting consequences of spinal cord injury (SCI), making improvements in bladder control one of the highest priorities for people with SCI [4]. Unfortunately, current treatment methods for people living with neurogenic bladder dysfunction, particularly catheters, only address symptoms and routinely cause urinary tract infections requiring hospitalization [5, 6].

Electrical stimulation of the nervous system offers the potential to address the underlying causes of neurogenic bladder dysfunction and recent studies of epidural spinal cord stimulation (SCS) accompanied by locomotor training have shown improvements in bladder function [7–9]. In fact, in humans, locomotor training alone improved bladder control [10], while SCS alone in rats with SCI modulated urethral sphincter activity [11]. Improvements in LUT control may therefore be driven either indirectly or through direct electrical recruitment of afferents innervating the bladder. In the case of limb motion, cervical and lumbar SCS can recruit muscles of the upper and lower limbs [12, 13] by activating reflexes [14, 15], demonstrating that focal stimulation of the afferent system can facilitate motor behaviors [16, 17].

Electrical stimulation of the pelvic and pudendal nerves can produce bladder contractions through a variety of reflex mechanisms [18–22]; stimulating afferents in the pudendal nerve can evoke reflexive micturition or suppress ongoing bladder contractions [21, 23, 24], while afferent activity in the pelvic nerve can modulate this reflex response [25]. These complex reflexes arise in part due to the multiple peripheral targets of the pudendal nerve. The pudendal nerve divides distally into the sensory, deep perineal, and caudal rectal branches [22]. The caudal rectal branch innervates the external anal sphincter and pelvic floor [26], while the sensory and deep perineal branches innervate the genitalia and urethra. Stimulation of these branches can either reflexively inhibit or evoke micturition [18, 22]. However, accessing and instrumenting these nerves could be challenging in humans and will require new surgical procedures, complicating translation of a peripheral nerve-based device [27–30].

In this study we sought to determine whether epidural SCS can selectively activate the peripheral nerve pathways that control the LUT. We tested this idea using custom epidural SCS electrode arrays, which are high-density relative to traditional clinical leads, to maximize the opportunity to activate focal sensory inputs to the spinal cord while minimizing recruitment of unwanted reflexes. Lower-limb activation frequently accompanies stimulation at the lumbosacral cord [31], potentially arising from the design of existing SCS arrays that often cover an entire segment of the spinal cord with just a few electrodes [32] and the diffuse fields that are generated by such electrodes. If selective activation were possible, this would establish epidural SCS as a method to directly modulate LUT function by activating sensory afferents in the pudendal and pelvic nerves.

## 2. Material and methods

### 2.1. Surgical procedures

Acute experiments were conducted under isoflurane anesthesia in six adult male cats weighing between 4.1 and 6.4 kg. All procedures were approved by the University of Pittsburgh Institutional Animal Care and Use Committee. All procedures were performed in accordance with the relevant guidelines and regulations.

The animals were anesthetized with a ketamine/acepromazine cocktail and anesthesia was maintained using isoflurane (1%–2%). A tracheostomy was performed and the trachea was cannulated and connected to an artificial respiration system. Throughout the procedure, the animal was artificially ventilated at 12–14 breaths per minute. Blood pressure was monitored with a catheter placed in the carotid artery. Temperature was maintained with a warm air heating pad and IV fluids were administered continuously. End tidal CO_2_, SpO_2_, core temperature, heart rate and blood pressure were monitored throughout the procedure and kept within a normal physiological range. Following experimental data collection, animals were euthanized with an IV injection of Euthasol.

**Figure 1.**
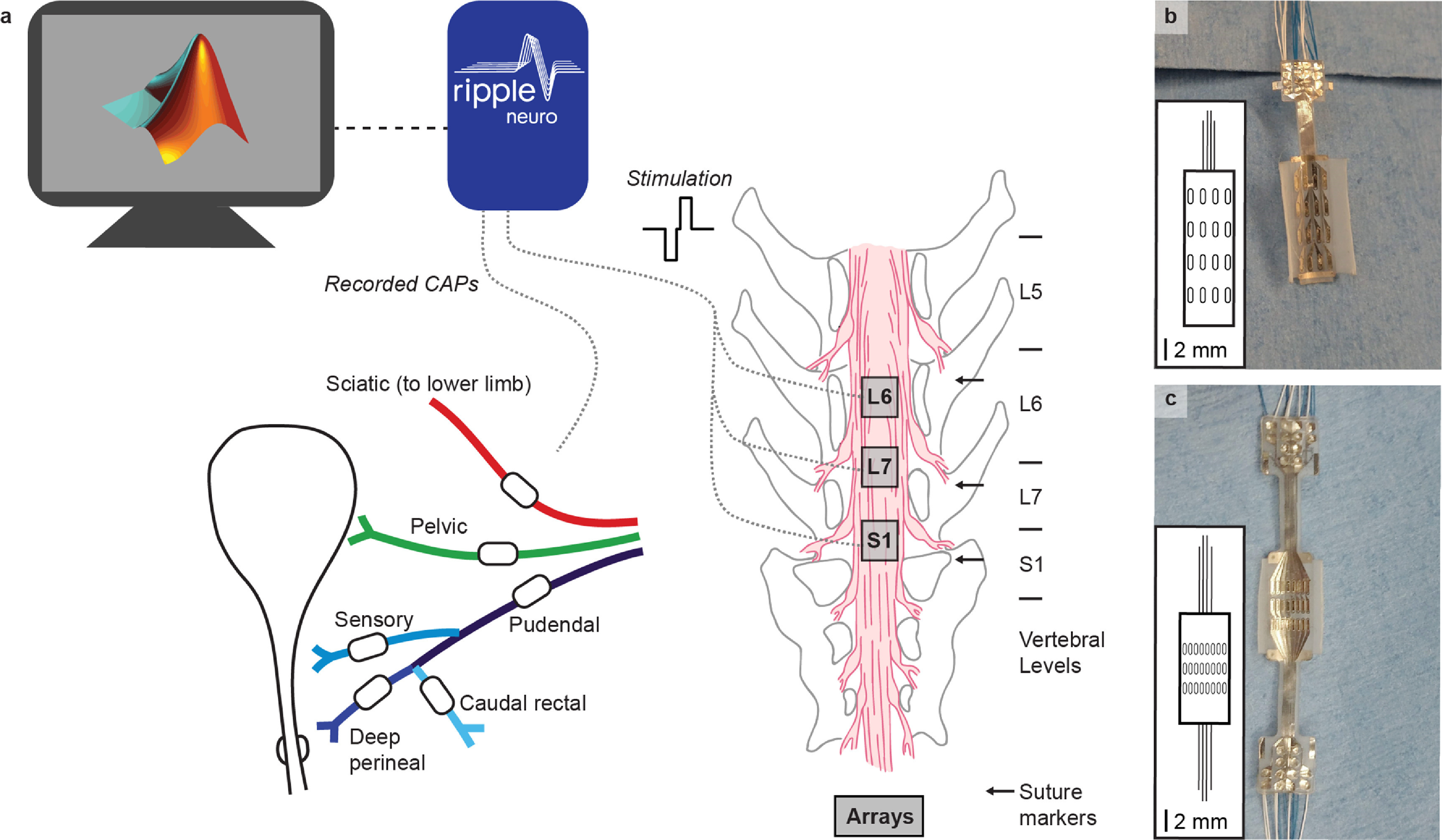
Experimental setup. (a) Nerve cuffs, shown as white ovals on each nerve, were placed on multiple peripheral nerves and a high-density electrode array was placed at three locations over the sacral cord and cauda equina. Suture markers were placed at the center of the dorsal spinal processes prior to the laminectomy and are labeled with arrows. Nerve cuffs on the pelvic nerve (green), pudendal nerve (blue), and pudendal branches (shades of blue) had an inner diameter between 500 *μ*m and 1000 *μ*m. The sciatic nerve (red) cuff had an inner diameter of 3 mm. Recording and stimulation were completed through a MATLAB interface with a Ripple Grapevine system using a closed-loop response detection algorithm. (b) In animals 1 and 2, a 16 channel epidural array with four electrode columns spaced laterally across the cord and four electrode rows spaced rostrocaudally was used. The inset shows the array layout to scale, with the wire bundle represented in the same orientation as the photo. The electrodes on the 16 channel array were each 0.45 mm × 1.35 mm and were spaced 0.69 mm apart laterally and 1.64 mm apart rostrocaudally. (c) In animals 3–6, a 24 channel epidural array with eight columns and three rows was used. The electrodes on the 24 channel array were each 0.29 mm × 1.0 mm and were spaced 0.23 mm apart laterally and 0.78 apart rostrocaudally.

To measure antidromic compound action potentials in afferent axons evoked by SCS, we placed bipolar nerve cuffs (Micro-Leads Inc., Somerville, MA) on the left pelvic and pudendal nerves as well as pudendal nerve branches (figure 1(a)). The pelvic nerve was dissected free near the internal iliac artery and a cuff was placed prior to the branching of the pelvic plexus. To access the pudendal nerve, we made an incision on the left hindquarters between the base of the tail and the ischial tuberosity. We then placed nerve cuffs on the left pudendal nerve and the sensory, deep perineal, and caudal rectal branches [22] of the left pudendal nerve. We also placed a five-pole spiral nerve cuff (Ardiem Medical, Indiana, PA) on the left sciatic nerve to measure off-target effects associated with the lower-limb. A recording reference electrode, consisting of a stainless steel wire with ∼1 cm of insulation removed, was placed subcutaneously in the left lower back.

The bladder was exposed through a midline abdominal incision and a dual-lumen catheter (Model CDLC-6D, Laborie Medical Technologies, Williston, VT) was placed through the bladder dome to infuse and withdraw fluids as well as to measure bladder pressure during the initial evaluation of pelvic nerve cuff placement. The catheter was secured in place with a purse string suture. During experiments, this catheter was used to continuously drain the bladder. Bladder pressures were not evaluated during stimulation, as stimulation frequencies were not designed to elicit bladder contractions, and the use of isoflurane anesthesia typically suppresses LUT reflexes [33].

We performed a laminectomy at the L6, L7, and S1 vertebral levels to expose the sacral spinal cord and cauda equina, then placed a custom epidural spinal cord array (Micro-Leads Inc., Somerville, MA) with 16 or 24 channels (figures 1(b) and (c)) on the spinal cord at three different locations (figure 1(a)). The electrodes on the 16 channel array were each 0.45 mm × 1.35 mm and were spaced 0.69 mm apart laterally and 1.64 mm apart rostrocaudally (figure 1(b), inset shown to scale). The electrodes on the 24-channel array were each 0.29 mm × 1.0 mm and were spaced 0.23 mm apart laterally and 0.78 apart rostrocaudally (figure 1(c), inset shown to scale). The centers of the L6, L7 and S1 dorsal spinal processes were marked using a suture placed in paraspinal muscles prior to the laminectomy. The epidural arrays were then placed on the epidural surface of the spinal cord and aligned to these suture markers. For the most rostral location, the arrays were placed such that the most rostral electrode on the array was aligned with the center of the L6 dorsal process. After experiments were completed at this location, the electrode array was moved so that the most caudal electrode on the array was aligned with the L7 suture marker. For the final location, the most caudal electrode on the array was aligned with the S1 suture marker. We chose to standardize placement of the electrode arrays relative to the vertebral anatomy as efforts to align the arrays to a standard functional level are challenging because of variability in the location of spinal motoneuron pools relative to both vertebral and spinal root anatomy [34]. Nevertheless, this placement scheme ensured that the arrays spanned the sacral cord and cauda equina, with the L6 placement always being over the sacral cord and the S1 placement always being over the cauda equina. A stimulation return electrode, consisting of a stainless-steel wire with ∼1 cm of insulation removed, was placed outside the spinal column, near the L7 transverse process. Landmarks and dorsal root entry zones were verified postmortem.

Five animals were tested at three vertebral levels and one animal was tested at two vertebral levels (L6 and S1), giving a total of 17 sets of data. In total, stimulation was delivered at 368 electrode sites.

### 2.2. Neural recording and stimulation

Stimulation was delivered with a Grapevine Neural Interface Processor through a Nano 2+ Stim high-current headstage (Ripple LLC), with stimulation patterns commanded from MATLAB (MathWorks Inc. Natick, MA). This headstage delivers stimulation current amplitudes of up to 1.5 mA and has a compliance voltage of ±8.5 V. The stimulation amplitude across all trials ranged from 10 to 1500 *µ*A with a resolution of 10 *µ*A between steps up to 1280 *µ*A, and a resolution of 20 *µ*A from 1280 to 1500 *µ*A. Stimulation pulses were symmetric with 200 *µ*s cathodal and anodal phases. Phases were separated by a 66 *µ*s interphase interval. For animals 1–2 and 5–6, the cathodal phase was applied first, followed by the anodal phase. For animals 3–4, the anodal phase was applied first due to a change in the software used to control stimulation. Discounting one animal with significantly higher thresholds (animal 2), the median detected thresholds for all animals were between 430 and 500 *μ*A. Regardless of which phase was applied first, the recruitment thresholds were no different (*p* = 0.78, Wilcoxon test).

Compound action potentials were sampled at 30 kHz with a Surf S2 headstage (Ripple LLC) through the Grapevine Neural Interface Processor. The signal was filtered with a high-pass filter with a 0.1 Hz cutoff followed by a low-pass filter with a 7.5 kHz cutoff, using 3rd order Butterworth filters. The signals from each pole of the bipolar nerve cuffs were then differenced to find the response on a given nerve.

### 2.3. Compound action potential detection

Stimulation artifacts were removed from the nerve cuff recordings by linearly interpolating between the sample immediately before the onset of each stimulus pulse to 0.5 ms after the end of each stimulus pulse. We then band-pass filtered the signal between 200 Hz and 3000 Hz using a 1st order Butterworth filter, applied first forward, then backward. The signal-to-noise ratio for detecting antidromic action potentials at the recruitment threshold is substantially less than one due to the presence of spontaneous activity in the nerves as well as general recording noise. Therefore, we used stimulus-triggered averaging to detect responses evoked by stimulation. In some cases, large responses occurred at sporadic intervals during the course of the stimulation train, and we first eliminated these responses to prevent them from affecting the average signal. We identified any responses which had three or more consecutive samples that were more than four standard deviations from the overall signal mean and removed them from the dataset. The median percent of responses removed was 0.6%. In 2 of 4494 trials, these large responses occurred more than 20% of the time and were not removed from the dataset. In animal 2, the recording noise on the pudendal nerve cuff was higher than expected, so we determined the threshold for this nerve manually.

The presence of a compound action potential on each nerve was determined by comparing responses following stimulation to baseline recordings in which no stimulation occurred, using a previously-published method [35, 36]. Baseline recordings were collected prior to stimulation on each individual channel. We divided the filtered baseline signal into segments that were the same length as the interpulse interval and calculated the root mean squared amplitude for both the baseline and signal samples using a sliding window that was 250 *μ*s long, using a 25 *μ*s step size. To determine the response detection threshold, we then calculated the mean and standard deviation of the root mean squared baseline amplitude. We repeated the calculation of the mean and standard deviation 200 times from a random subsample of 80% of the baseline recording segments, and found the 99th percentile of the mean baseline. We then set the detection threshold to four standard deviations above the upper bound of this baseline mean, or a minimum of 0.5 *μ*V. This threshold was determined empirically to detect true responses without false positives. The stimulus-triggered average was calculated 200 times from a random subsample of 80% of the responses in order to find a distribution of typical responses. In each of these responses, the root mean squared amplitude of each time window was compared to the root mean squared of the baseline amplitude. If 95% of these responses were suprathreshold and nerve activity was detected for at least three consecutive windows, the response was considered significant.

### 2.4. Determining recruitment thresholds

In the first experiment, we tested a fixed set of amplitudes between 150 *µ*A and 800 *µ*A at increments of 50 *µ*A. In the remaining experiments, we used a binary search procedure to determine the minimum stimulus current necessary to recruit each nerve according to methods published previously [35, 36]. First, we delivered 50 stimulation pulses through each electrode on the array in a random order, using high amplitude pulses at 20 Hz and switching electrodes after each pulse, to determine which electrodes could evoke responses in the peripheral nerves. The stimulation amplitude for this trial was determined based on the highest amplitude that did not evoke substantial movement in the leg, or when all electrodes showed neural responses, and typically ranged from 600 to 1000 *µ*A. After the responses to stimulation at the maximum amplitude were determined, all stimulation electrodes that evoked compound action potentials in at least one instrumented nerve were tested individually using a binary search procedure to determine the thresholds for every nerve recruited. We set the stimulation frequency during these trials by determining the longest-latency neural response on each nerve cuff, adding 5 ms, and taking the inverse of this time. In practice, this resulted in stimulation frequencies ranging from 25 to 100 Hz. With this approach, we were able to maximize the stimulation frequency and minimize overall experiment time. Three hundred stimulation pulses were delivered to each electrode at each tested amplitude. For each nerve showing a response, we determined the current threshold to a resolution of 10 *µ*A. This procedure typically took 2–3 h for each location of the spinal cord array.

To determine the selectivity of this high-density epidural SCS array, we measured the recruitment thresholds, selectivity, and dynamic range of stimulation-evoked neural responses. Recruitment thresholds for each nerve were defined as the lowest amplitude at which a response to SCS was detected. Threshold responses were considered to be selective when only a single nerve responded at the threshold amplitude and non-selective when multiple nerves were simultaneously recruited at the threshold amplitude. We determined whether pudendal nerve branches were activated selectively by excluding the pudendal nerve and comparing their recruitment thresholds only to other branches, the pelvic nerve, and the sciatic nerve. We defined the dynamic range of stimulation on an electrode as the difference between the threshold amplitude for the recruited nerve and the first higher amplitude at which multiple nerves were recruited.

### 2.5. Statistics

The recruitment thresholds were not normally distributed (*p* < 0.001, Lilliefors test) so data are reported as the median threshold amplitude with lower and upper quartiles (IQR). The Wilcoxon rank-sum test was used to test for differences between two groups. For comparisons between multiple groups of data, we used a Kruskal–Wallis test with a Dunn’s test for post-hoc analysis. The data were analyzed in Matlab 2018a (Mathworks, Natick, MA).

## 3. Results

To test whether SCS could recruit peripheral nerves that innervate the LUT, we stimulated through each contact on the electrode arrays while they were positioned over the sacral spinal cord and cauda equina (figure 1). We measured antidromic recruitment of afferents in the pelvic, pudendal and sciatic nerves, and used the relative recruitment of these nerves to determine selectivity in six anesthetized cats. These pathways are co-activated in normal function [18, 37] so we also measured the co-recruitment of all these nerves at increasing stimulation amplitudes.

### 3.1. High-density SCS selectively recruits pelvic and pudendal afferents

**Figure 2.**
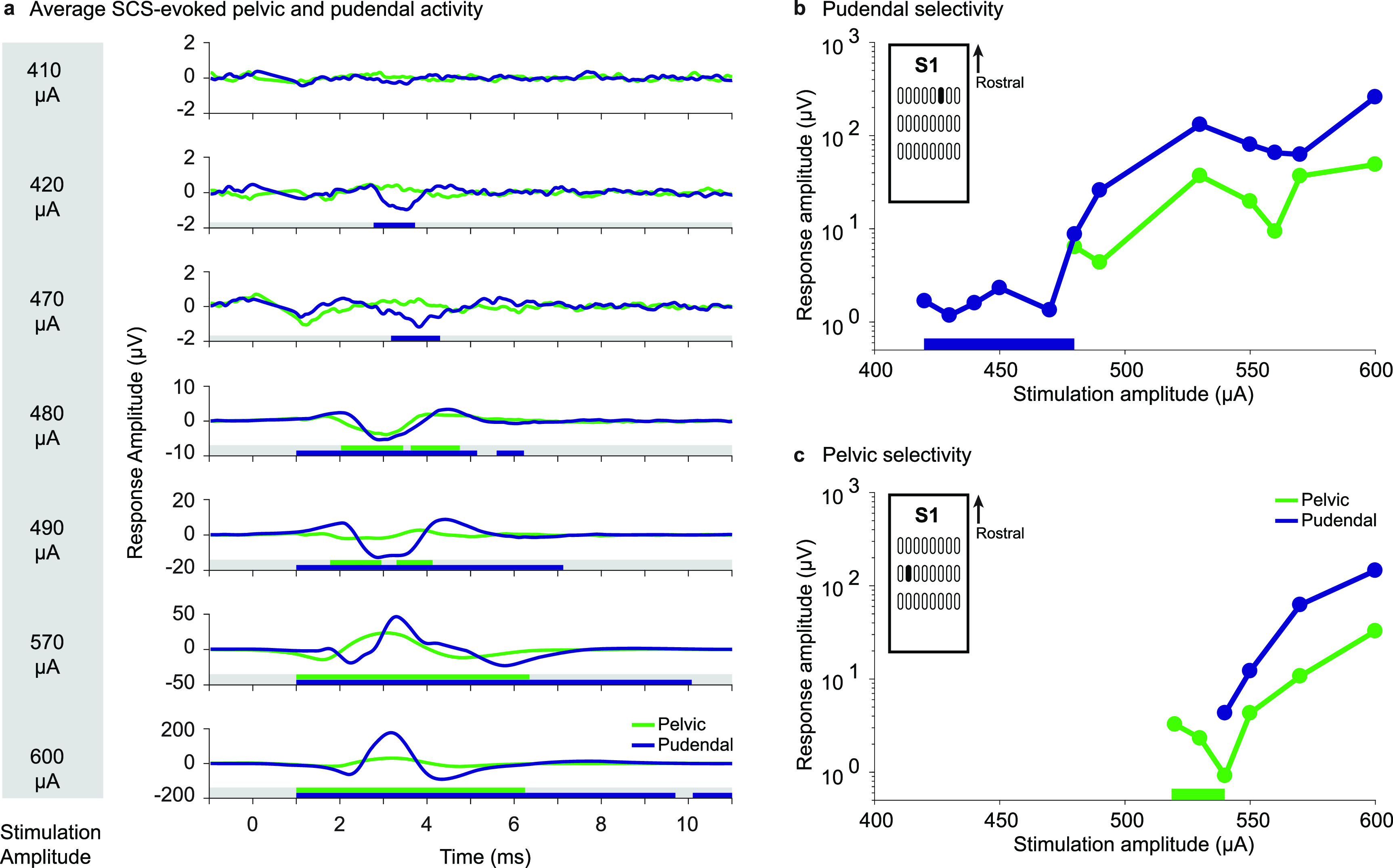
Examples of selective pelvic and pudendal nerve recruitment in animal 3 during stimulation on two different electrodes. (a) Stimulation-triggered averages of the pelvic (green) and pudendal (purple) nerve compound nerve action potentials at selected stimulation amplitudes. The traces include 1 ms preceding the stimulus pulse. Windows in which responses were detected are indicated under each trace as colored bars. At 410 *μ*A, no response was detected in either nerve. At 420 *μ*A, a selective response was detected in the pudendal nerve. At 480 *μ*A the pelvic nerve was also recruited. 600 *μ*A was the maximum stimulation amplitude for this trial and evoked large compound action potentials in both nerves. Note the different *y*-axis scales for each stimulation amplitude. (b) Peak-to-peak compound action potential amplitude of the pelvic and pudendal nerves for the electrode illustrated in (a) that was selective for the pudendal nerve from 420 *μ*A up to 480 *μ*A (selective range highlighted with a bar along the *x*-axis). Only the pudendal and pelvic nerve traces are shown here, but this electrode did not recruit any other instrumented nerves at threshold. The *y*-axis is shown on a log scale. The specific stimulation electrode is highlighted in the inset. (c) Peak-to-peak compound action potential amplitude of the pelvic and pudendal nerves for a nearby electrode (see inset) that selectively recruited the pelvic nerve from 520 *μ*A up to 540 *μ*A (selective range highlighted with a bar along the *x*-axis). The *y*-axis is shown on a log scale.

High-density epidural SCS selectively recruited both the pelvic and the pudendal nerves at the sacral cord and cauda equina in all but one animal. Surprisingly, we found that individual electrodes within the array could selectively recruit different nerves even though the electrodes were often very close together. As a typical example (animal 3, figure 2), an individual electrode selectively recruited the pudendal nerve at 420 *μ*A and the pelvic nerve was not recruited until the amplitude was increased to 480 *μ*A (figures 2(a) and (b)). A nearby electrode recruited the pelvic nerve selectively at 520 *μ*A (figure 2(c)).

**Figure 3.**
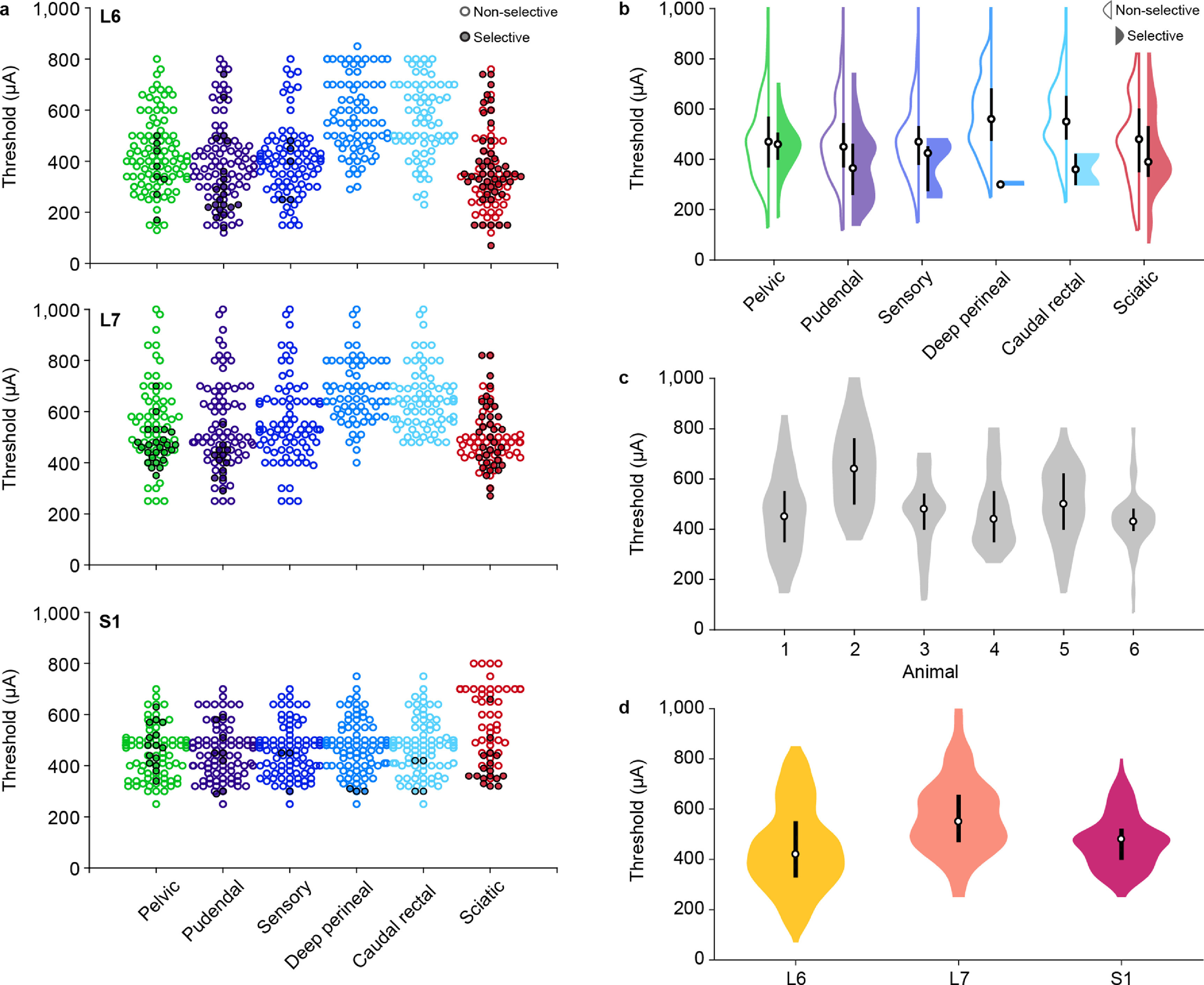
Recruitment thresholds for all animals, nerves and locations. (a) Recruitment thresholds for each nerve at each location across all animals. Trials that recruited nerves non-selectively at the threshold amplitude for that nerve are marked with unfilled circles and trials that recruited nerves selectively at the threshold amplitude are marked with filled circles. (b) Recruitment thresholds for each nerve across all locations and animals. Trials that recruited nerves non-selectively are marked with unfilled violins, left, and trials that recruited nerves selectively are marked with filled violins, right. (c) Recruitment thresholds for each animal across all nerves and locations. (d) Recruitment thresholds for each location across all nerves and animals. In the violin plots, the circle indicates the median value and the thick vertical bar indicates the interquartile range.

We characterized nerve recruitment in three ways: selective recruitment at the threshold amplitude, total recruitment at the threshold amplitude, and recruitment at the maximum stimulation amplitude. Overall, the pelvic and pudendal nerves were both selectively and non-selectively recruited at all levels (figure 3(a)). The pelvic nerve was selectively recruited between 170 and 700 *μ*A, while these thresholds were between 140 and 740 *μ*A for the pudendal nerve. There was a difference between the median threshold amplitudes required to selectively recruit these nerves (pelvic: 460 *μ*A, pudendal: 365 *μ*A, *p* < 0.005, Wilcoxon test, figure 3(b), filled violins). There was no difference in the threshold amplitude when the pelvic and pudendal nerves were not recruited selectively (*p* = 0.71, Wilcoxon test) and ranged between 120 and 1000 *μ*A (figure 3(b), unfilled violins). While there was no difference in the recruitment thresholds between the pelvic and pudendal nerves, when all nerves were considered, there was a significant difference in the threshold amplitudes across animals (*p* < 0.001, Kruskal–Wallis test, figure 3(c)) and spinal locations (*p* < 0.001, Kruskal–Wallis test, figure 3(d)). At the level of the L6 and S1 vertebrae, the threshold amplitudes were lower than with the arrays placed at the L7 vertebra (*p* < 0.001, Dunn’s test, figure 3(d)) and placing the arrays at the L6 vertebra resulted in lower threshold amplitudes than when they were placed under the S1 vertebra (*p* < 0.005, Dunn’s test).

**Figure 4.**
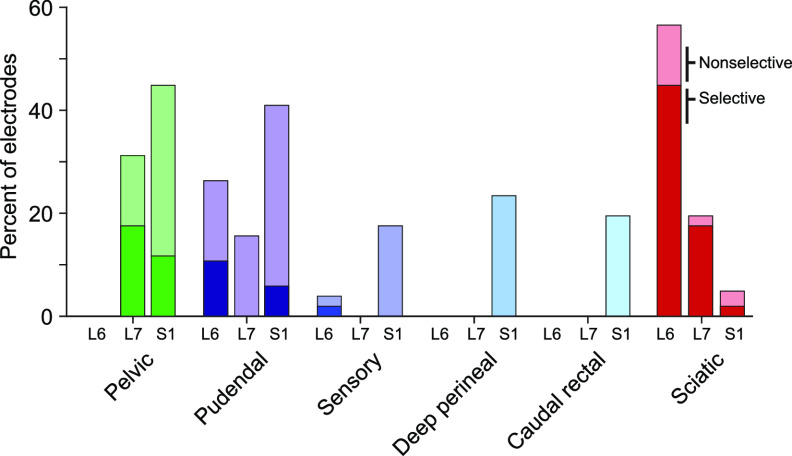
Recruitment of all nerves at threshold at each spinal level. Selective and nonselective nerve recruitment at each location at the threshold amplitude. The darkest color (bottom) in each stacked bar is the median percentage of electrodes that recruited each nerve selectively at the threshold amplitude. The lighter shade (top) represents the percentage of electrodes that recruited each nerve non-selectively at the threshold amplitude. Thus, the cumulative total of the bars represents the total recruitment of each nerve at the threshold amplitude.

The pelvic and pudendal nerves were both recruited selectively in all but one animal (animal 6). The pelvic nerve was recruited selectively at 10 of the 14 tested array locations across the remaining five animals. Selective recruitment of the pelvic nerve occurred most consistently when the array was at the level of the S1 dorsal process (five animals) and occurred on 12.5% of the electrodes at this level (figure 4, dark green bars, table 1). The pelvic nerve was also recruited selectively in two animals at the L6 vertebral level and in three animals at the L7 vertebral level (table 1).

**Table 1.** Selective recruitment of each nerve in all animals by vertebral level. The percentage of electrodes at a given array location that selectively recruited each nerve are shown as the median and upper and lower quartiles. The number of animals where stimulation at each array location selectively recruited each nerve is also shown.

	L6 array placement		L7 array placement		S1 array placement	
Nerve	% of electrodes	# of animals	% of electrodes	# of animals	% of electrodes	# of animals
Pelvic	0.0 (0.0–4.2)	2/6	18.8 (0.0–35.4)	3/5	12.5 (8.3–16.7)	5/6
Pudendal	11.5 (0.0–20.8)	4/6	0.0 (0.0–14.6)	2/5	6.3 (0.0–12.5)	4/6
Sensory	2.1 (0.0–4.2)	3/6	0.0 (0.0–0.0)	0/5	0.0 (0.0–0.0)	1/6
Deep perineal	0.0 (0.0–0.0)	0/6	0.0 (0.0–0.0)	0/5	0.0 (0.0–0.0)	1/6
Caudal rectal	0.0 (0.0–0.0)	0/6	0.0 (0.0–0.0)	0/5	0.0 (0.0–0.0)	1/6
Sciatic	47.9 (20.8–50.0)	6/6	18.8 (9.4–34.4)	5/5	2.1 (0.0–8.3)	3/6

The pudendal nerve was recruited selectively at 11 of the 14 tested array locations across the five animals in which selective recruitment occurred. Selective recruitment occurred most often with the array at the L6 vertebra (four animals) and occurred on 11.5% of the electrodes at this level (figure 4, dark purple bars). In four animals the pudendal nerve was recruited selectivity at the S1 level, and in two animals the pudendal nerve was recruited selectively at the L7 level (table 1).

On some SCS electrodes the threshold stimulation amplitude evoked activity in multiple peripheral nerves simultaneously. Therefore, we also measured the combined selective and non-selective recruitment at the threshold amplitude. We also measured nerve recruitment through SCS electrodes at high amplitudes (well above the threshold amplitude) to characterize the maximum recruitment potential of an individual electrode. The pelvic nerve was recruited at threshold on 30.7% of the electrodes across all placements (see figure 4 and table 2 for additional detail) and at the maximum stimulation amplitude on 73.7% of the electrodes (see table 3 for additional detail). The pudendal nerve was recruited at threshold on 31.5% of the electrodes across all placements (see figure 4 and table 2 for additional detail) and at the maximum amplitude on 75.3% of electrodes (see table 3 for additional detail). On 16.7% of the electrodes, or about 4 electrodes on a 24-channel array, stimulation at maximal intensities evoked no detectable response on either the pelvic or pudendal nerves.

**Table 2.** Total threshold recruitment of each nerve in all animals by vertebral level. The percentage of electrodes at a given array location that recruited each nerve selectively or non-selectively at threshold are shown as the median and upper and lower quartiles. The number of animals where stimulation at each array location recruited each nerve is also shown.

	L6 array placement		L7 array placement		S1 array placement	
Nerve	% of electrodes	# of animals	% of electrodes	# of animals	% of electrodes	# of animals
Pelvic	0.0 (0.0–41.7)	2/6	33.3 (0.0–76.6)	3/5	47.9 (33.3–68.8)	6/6
Pudendal	28.1 (8.3–45.8)	5/6	16.7 (0.0–56.8)	4/5	43.8 (37.5–75.0)	6/6
Sensory	4.2 (0.0–8.3)	4/6	0.0 (0.0–18.8)	2/5	18.8 (12.5–81.3)	5/6
Deep perineal	0.0 (0.0–0.0)	1/6	0.0 (0.0–0.0)	0/5	25.0 (8.3–37.5)	5/6
Caudal rectal	0.0 (0.0–0.0)	1/6	0.0 (0.0–0.0)	0/5	20.8 (12.5–31.3)	5/6
Sciatic	60.4 (41.7–62.5)	6/6	20.8 (14.1–42.7)	5/5	5.2 (0.0–8.3)	4/6

**Table 3.** Maximum amplitude recruitment of each nerve in all animals by vertebral level. The percentage of electrodes at a given array location that recruited each nerve at the maximum amplitude are shown as the median and upper and lower quartiles. The number of animals where stimulation at each array location recruited each nerve is also shown.

	L6 array placement		L7 array placement		S1 array placement	
Nerve	% of electrodes	# of animals	% of electrodes	# of animals	% of electrodes	# of animals
Pelvic	83.3 (62.5–91.7)	6/6	87.0 (56.3–92.2)	5/5	64.6 (62.5–100.0)	6/6
Pudendal	81.3 (70.8–91.7)	6/6	83.3 (56.3–92.2)	5/5	72.9 (66.7–100.0)	6/6
Sensory	75.0 (62.5–91.7)	6/6	83.3 (46.9–93.8)	5/5	70.8 (62.5–100.0)	6/6
Deep perineal	66.7 (41.7–83.3)	6/6	70.8 (46.9–84.4)	5/5	66.7 (62.5–100.0)	6/6
Caudal rectal	63.5 (29.2–87.5)	6/6	83.3 (43.8–92.2)	5/5	64.6 (58.3–100.0)	6/6
Sciatic	81.3 (62.5–91.2)	6/6	83.3 (46.9–93.8)	5/5	81.3 (25.0–81.3)	6/6

### 3.2. Recruitment of pudendal nerve branches

Activating different branches of the pudendal nerve can have different effects on bladder function [22, 24], and we were therefore interested in determining the recruitment properties of these individual branches (sensory, deep perineal, and caudal rectal). SCS evoked responses in every branch of the pudendal nerve in every cat at the maximal stimulation amplitude (table 3). There was no difference in the number of electrodes that could recruit these nerves at different vertebral levels at these high intensities (*p* = 0.90, Kruskal–Wallis test).

Pudendal nerve branches were recruited at the threshold amplitude most often at the S1 location (figure 4, blue bars). At this location, all three branches were recruited at threshold in five out of six animals (table 2) and the caudal rectal and deep perineal nerves were significantly more likely to be recruited at threshold compared to other locations (*p* < 0.05, Dunn’s test), although this difference was not significant for the sensory branch (*p* = 0.11, Kruskal–Wallis test).

Selective recruitment of the pudendal nerve branches was rare in all animals at all spinal locations (table 1). The sensory branch was recruited selectively at just four of the 17 total tested locations. The other two branches were each selectively recruited at only one of the 17 locations, with the deep perineal branch recruited selectively on just three electrode contacts across all experiments and the caudal rectal branch recruited selectively on just four contacts (figure 3(a)).

Lastly, the stimulation amplitude required to recruit the deep perineal and caudal rectal nerves was significantly higher than all other nerves at the L6 and L7 vertebral levels (*p*< 0.001, Dunn’s test, figure 3(a)). This was not true at the S1 vertebral level, where there was no difference in the recruitment threshold for any LUT nerve (*p* = 0.59, Kruskal–Wallis test, figure 3(a)).

### 3.3. Sciatic nerve recruitment

A common side effect of electrically stimulating the sacral cord or nerves is lower limb movement resulting from sciatic nerve activation [7, 11, 38, 39]. Therefore, we monitored sciatic nerve activity during these experiments and found that similar to the results for the pelvic and pudendal nerves, SCS activated the sciatic nerve both selectively and non-selectively at all levels. The sciatic nerve had lower thresholds than all nerves except for the pudendal nerve and its sensory branch at the L6 location (*p* < 0.05, Dunn’s test, figure 3(a)). The sciatic nerve had lower thresholds than the deep perineal and caudal rectal branches at the L7 location (*p* < 0.001, Dunn’s test, figure 3(a)). Conversely, sciatic threshold amplitudes were higher than the pelvic and pudendal nerves at the S1 lamina location (*p* < 0.005, Dunn’s test, figure 3(a)). At that location, sciatic threshold amplitudes were also higher than the sensory and caudal rectal branches of the pudendal nerve (*p* < 0.05, Dunn’s test, figure 3(a)), but were not significantly different than the deep perineal nerve (*p* = 0.054, Dunn’s test, figure 3(a)).

These lower thresholds compared to LUT nerves are likely responsible for the fact that at the L6 and L7 laminar locations, the sciatic nerve was recruited selectively more often than any LUT nerve (figure 4, dark red bars). However, at the S1 location, there was no difference in the number of electrodes that selectively recruited LUT and sciatic nerves (*p* = 0.13, Kruskal–Wallis test).

### 3.4. Nerve selectivity changes with stimulation location

**Figure 5.**
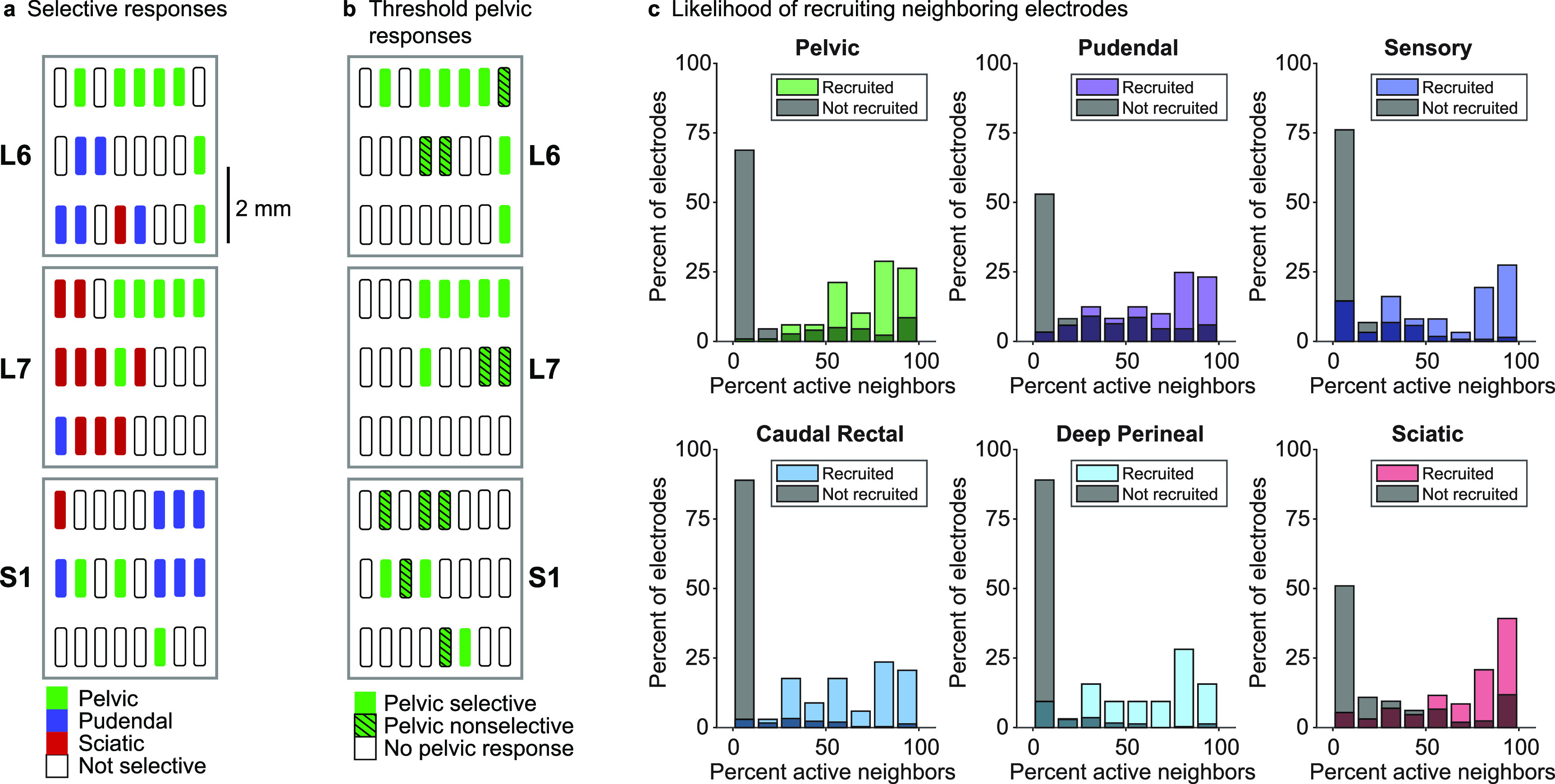
Spatial arrangement of evoked responses. (a) Selective recruitment for the pelvic nerve, pudendal nerve, and sciatic nerve in a representative animal (animal 3). (b) Pelvic nerve recruitment for the same animal as panel a, demonstrating that the pelvic nerve was frequently recruited non-selectively on electrodes adjacent to selective electrodes. (c) When an electrode activated a particular nerve at the threshold amplitude, neighboring electrodes were likely to activate that nerve as well (colored bars). The *y*-axis is normalized to the total number of electrodes that activated a specific nerve. When that nerve had not been activated, surrounding electrodes were much less likely to activate neighboring electrodes (gray bars). The *y*-axis for the gray bars is normalized to the total number of electrodes that did not activate a specific nerve.

In most animals the pelvic, pudendal, and sciatic nerves were selectively recruited by multiple electrodes. We investigated the extent to which these patterns of recruitment tended to be organized within the array. Figure 5(a) shows a representative example of the nerves selectively recruited by individual electrodes across the three array levels in one animal. Many electrodes recruited nerves selectively (figure 5(a), colored rectangles), while other electrodes only recruited nerves non-selectively (figure 5(a), white rectangles). No obvious organization of nerve recruitment was seen when we considered only purely selective electrodes. However, when we examined both selective and non-selective responses at threshold, we observed stimulation ‘hot spots’ within the arrays (figure 5(b)). To quantify similarities in spatial recruitment we asked whether stimulation on a given electrode recruited the same nerves as its neighbors. When we stimulated through an electrode that activated a particular nerve at threshold, 75.0% (IQR 42.9%–87.5%) of the neighboring electrodes recruited that same nerve at threshold. Details for individual nerves are shown in figure 5(c) (colored bars). Conversely, if an electrode did not recruit a nerve at threshold, the neighboring electrodes were also unlikely to recruit that nerve at threshold, with a median recruitment of 0.0% (IQR 0.0%–14.3%) (figure 5(c), gray bars). Every nerve was recruited more frequently at threshold when it was also recruited at threshold on an adjacent electrode (figure 5(c), colored bars).

Additionally, because only nerves on the left side of the animal were instrumented, we examined the threshold amplitudes required for SCS electrodes on ipsilateral (left) or contralateral (right) side of the cord to recruit ipsilateral nerves. For the 4-column electrode, we compared the median thresholds for the most ipsilateral electrode column to the most contralateral electrode column. For the 8-column electrode, we compared the median thresholds for the two most ipsilateral columns to the two most contralateral columns. At all spinal levels, the difference in threshold amplitudes between the ipsilateral and contralateral sides was significant (*p* < 0.001, Wilcoxon test). At L6, the median recruitment amplitudes for contralateral electrodes were 1.5 (range 1.0–2.7) times larger than those for ipsilateral electrodes. At L7, the median recruitment amplitudes for contralateral electrodes were 1.3 (range 1.3–1.5) times larger than those for ipsilateral electrodes. However, at S1, the median recruitment amplitude of the contralateral electrodes was 0.9 times that of ipsilateral electrodes. Although this effect was significant, in individual cats the difference in amplitudes between the two sides of the cord was inconsistent at S1, ranging from 0.7 to 1.2.

### 3.5. Nerve coactivation

Because we observed numerous electrode contacts with non-selective nerve recruitment, we wanted to quantify the extent to which this recruitment was limited to LUT nerves as compared to co-activation with the off-target sciatic nerve. This co-activation of different groups of nerves varied by level.

**Figure 6.**
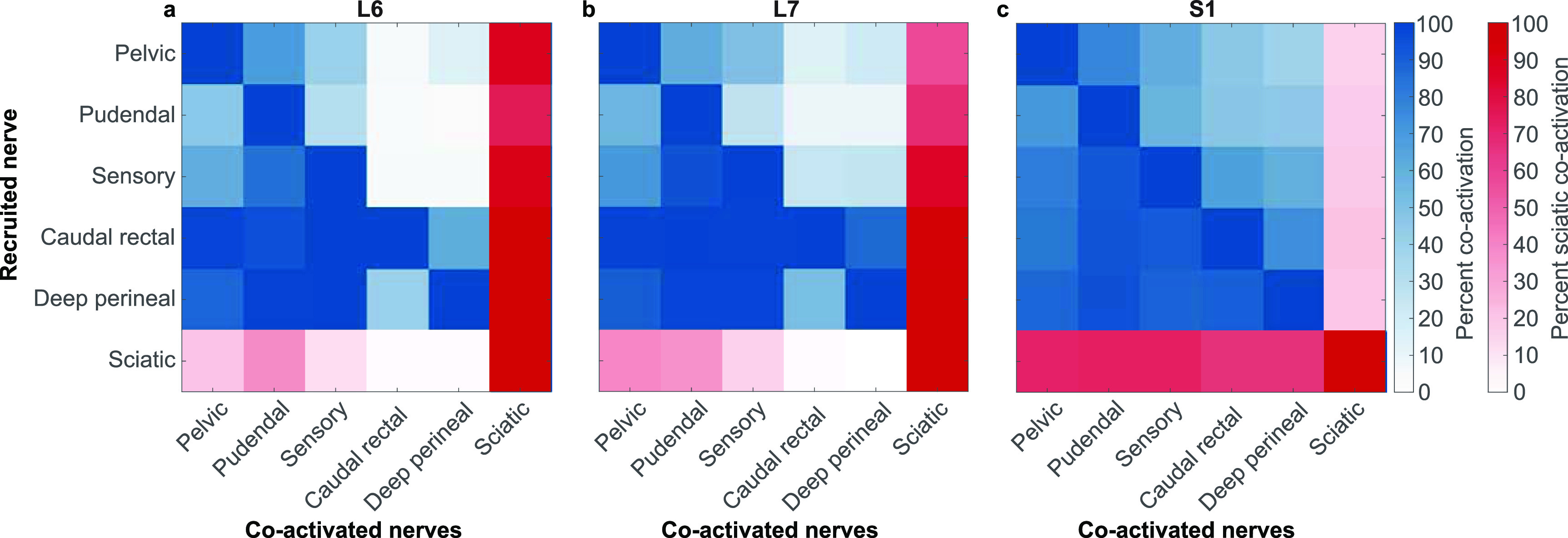
Coactivation of all nerves. Results are shown for each electrode placement at the (a) L6 vertebral segment, (b) L7 vertebral segment and (c) S1 vertebral segment. When a nerve first became active (vertical axis), other nerves were often co-activated or were recruited at lower amplitudes (horizontal axis). Sciatic comparisons are colored differently for clarity. As an example, in (a) when the pelvic nerve was first recruited, the sciatic nerve was frequently also active. However, when the sciatic nerve was first recruited, the pelvic nerve was less likely to be active.

The pelvic and pudendal nerves were more likely to be coactivated with the sciatic nerve at more rostral array placements (figures 6(a) and (b)). At the L6 level, the sciatic nerve was recruited with the pelvic nerve 87.4% of the time and with the pudendal nerve 74.8% of the time. At the L7 level this coactivation decreased to 57.5% for the pelvic nerve and 68.2% for the pudendal nerve. At the S1 level, coactivation decreased further still to 16.1% for the pelvic nerve and 18.4% for the pudendal nerve (figure 6(c)). The pelvic and pudendal nerves were coactivated more frequently at increasingly caudal spinal levels (figure 6). When the pudendal nerve was recruited, the pelvic nerve was also active 46.6%, 56.8% and 70.4% of the time at the L6, L7, and S1 locations, respectively.

### 3.6. Dynamic range of recruited nerves

**Figure 7.**
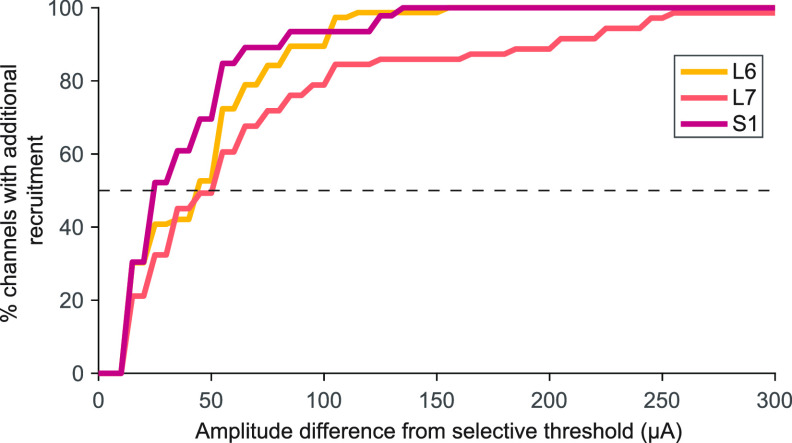
Distribution of dynamic ranges for each array location. The dynamic range of each selective electrode is the amount of additional stimulation current necessary to evoke activity in an additional nerve, over and above the initial selective response. Many nerves recruited selectively had a dynamic range of less than 50 *μ*A. Median dynamic range is marked with a dotted line.

While the primary aim of this experiment was to identify whether LUT afferents could be recruited selectively by SCS, we also wanted to characterize the dynamic range of stimulation on each electrode. The dynamic range is the stimulus amplitude range between the threshold amplitude and the stimulation amplitude at which additional nerves are recruited. Within this range, stimulation remains selective. The dynamic range was different at different vertebral levels (*p* < 0.001, Kruskal–Wallis test, figure 7). The dynamic range was the lowest at S1 and had a median value of 20 *μ*A (IQR 10–50 *μ*A) across all electrodes and animals. With the array placed at the L6 vertebral level, the median dynamic range was 40 *μ*A (IQR 10–60 *μ*A), and at the L7 vertebral level, the median dynamic range was 45 *μ*A (IQR 20–80 *μ*A).

The dynamic range also varied between different nerve groups. The amplitude difference between LUT nerve recruitment and coactivation with other LUT nerves was 30 *μ*A (IQR 10–50 *μ*A). For LUT nerves to become coactivated with the sciatic nerve, the dynamic range was 20 *μ*A (IQR 10–50 *μ*A). Finally, when the sciatic nerve was recruited selectively, the dynamic range was 50 *μ*A (IQR 25–100 *μ*A) before recruiting LUT nerves.

## 4. Discussion

We found that epidural SCS over the sacral cord can recruit axons arising from the pelvic and pudendal nerves, providing a mechanism to directly modulate bladder function. While completely selective recruitment of LUT nerves was not common (figure 4), selective recruitment of the pelvic, pudendal, and sciatic nerves was possible at all three vertebral levels tested and in five of the six animals. In the sixth animal, there were no notable surgical or electrophysiological factors that we observed that would have led to this loss of selectivity. However, it is possible that small variations in electrode coupling to the dura or the nerve cuff recordings could have played a role.

Epidural SCS has been explored in combination with locomotor training to improve LUT control in humans [7] and rats [8]. However, it was unclear from these studies whether the benefits of stimulation on bladder function arose directly from SCS itself, or whether the benefits were primarily driven by indirect effects such as improved mobility. Our results demonstrate a physiological mechanism for SCS to directly recruit LUT reflexes and therefore modulate bladder function. While the anesthesia and stimulation frequencies used in this study were not designed to elicit actual bladder contractions, we believe that characterizing direct nerve recruitment through SCS is an important step to facilitate future work targeting bladder reflexes. In these experiments, both pelvic and pudendal nerves were activated by SCS, and stimulating pelvic nerve [40, 41] and pudendal nerve [18, 21–23] afferents can facilitate or inhibit micturition. While it might seem more obvious to directly target the pelvic and pudendal nerves, surgical access to these nerves can be challenging in humans, and the pelvic nerve is particularly inaccessible [42, 43]. SCS primarily activates afferent axons in therapeutic use [14], and while SCS can directly activate efferents at high stimulation amplitudes, at the threshold amplitude the activated fibers are likely composed exclusively of afferents. Here, we demonstrate that it is possible to access LUT afferent pathways using sacral SCS, although the degree to which it is important to selectively activate specific branches remains unclear.

We have further demonstrated that these high-density epidural spinal cord arrays are able to produce completely different recruitment patterns within the same spinal level simply by changing the active electrode within the array. This would be difficult to achieve with standard clinical leads, which typically have two to three contacts per segment. This result suggests that high-density epidural electrode arrays could be particularly beneficial to optimize SCS for improving bladder function—or any other function of interest at other spinal levels [44–49]—and that existing commercial stimulation leads may be inadequate.

We placed our stimulation electrodes over the sacral spinal cord rather than more rostral levels of the cord, where many clinical implants are placed [50–53] as the sacral cord contains the motoneuron pools of the bladder and urethral sphincter [34] and gives rise to the entirety of the pelvic and pudendal nerves [54–56]. While there were some differences between stimulation locations in terms of threshold amplitudes (figure 3(d)), selectivity (figure 4) and dynamic range (figure 7), these differences were subtle. In fact, within any given animal, we were able to recruit all instrumented nerves at all levels.

We evoked the most activity in pelvic afferents when the array was at more caudal locations while pudendal nerve afferents were recruited at more rostral locations (tables 1 and 2). This is consistent with previous anatomical observations of the roots that contribute to each of these nerves. The pudendal nerve is typically composed of fibers from the S1 and S2 roots, while the pelvic nerve is typically composed of fibers from the S2 and S3 roots [57, 58]. Afferents of the pelvic nerve may however be located more rostrally, in the S1 and S2 roots, in some animals [59]. Furthermore, motoneuron pools for muscles innervated by the pudendal nerve tend to be located in the S1 and S2 cord in Onuf’s nucleus, while motoneuron pools for muscles innervated by the pelvic nerve are typically focused in the S2 and S3 cord [34, 58].

### 4.1. Lower limb activation

If the sciatic nerve were always activated during stimulus trains intended to recruit LUT nerves, the associated lower-limb movement could be very disruptive. In fact, activation of the lower limb is a common problem with the commercially available InterStim sacral nerve stimulators [60] and the Finetech anterior root stimulation system [61]. Although motor activation of the lower-limb does not prevent bladder prostheses from being effective, it is typically an undesirable off-target effect [7, 11, 38, 39]. On the other hand, recruiting sensory afferents of the lower limb, particularly the tibial nerve, has been shown to improve continence [62], making this a potentially useful target in some contexts. We found that in the spinally intact cat, there was substantially less activation of the sciatic nerve when the electrodes were over the cauda equina compared to the sacral cord, which is consistent with the path of the lower lumbar and sacral roots within the spinal canal at these locations.

### 4.2. LUT co-activation

While this study focused on selective nerve activation, axons in many different LUT nerve branches are active simultaneously in behavior. For instance, the anal sphincter, innervated by the caudal rectal nerve, and the external urethral sphincter, innervated by the deep perineal nerve, are frequently coupled [37]. Further, in some cases, co-stimulation of multiple pudendal branches improves voiding efficiency [24]. It is therefore likely that selectively activating these branches may not be necessary to effectively control bladder function. This is encouraging because we found that the dynamic range for selective stimulation was typically less than 100 *µ*A.

The sensory branch typically had lower threshold amplitudes than the deep perineal and caudal rectal branches (figure 3(b)). Because SCS primarily recruits afferents, this difference in threshold could be due to the high density of afferent fibers in the sensory branch compared to both the deep perineal and caudal rectal branches, which have substantial motor functions [57].

### 4.3. Limitations

In this study we used monopolar stimulation exclusively, which allowed us to systematically test all the electrodes in the available time, but may have been suboptimal to recruit afferent populations selectively. To improve selectively, multipolar stimulation can be used to localize or focus current between several electrodes [63]. In a recent study in humans, multipolar stimulation was often required to evoke meaningful sensory percepts in amputees, while monopolar stimulation was generally less effective [64]. Similarly, some commercially available SCS systems leverage multipolar stimulation to increase the focality of the paresthesias evoked by stimulation [65]. Additional selectivity could potentially be gained by changing stimulation waveforms [66, 67] or applying variable-frequency stimuli [68].

Another limitation of this study is that it was conducted in acute experiments and we did not determine the stability of these effects during movement. Postural effects are known to be considerable in human SCS [51], and these effects could be exacerbated using small electrodes of the type considered here. Additionally, this study was performed in cats, and the reduced cerebrospinal fluid thickness in combination with the smaller cross-sectional area of the spinal cord in cats [69] compared to humans may impact parameter choice, particularly threshold amplitudes. The smaller spinal cord surface area in cats also requires fewer electrodes to cover the spinal cord, while array placement will differ due to the different number of lumbar segments and differences in alignment between the vertebrae and spinal segments in cats and humans [69]. The large number of electrodes that could be required in a human application might also require new methods of parameter tuning to be developed, including closed-loop methods where muscle activity or other non-invasively accessible signals are used to automatically tune parameters. Ultimately, the goal of this work is to manipulate LUT function, and here we only study peripheral nerve recruitment, particularly recruitment of the afferent fibers.

### 4.4. Implications for neuroprosthetic devices

This study demonstrates that it is possible to selectively activate individual peripheral nerves innervating the LUT with high-density SCS. This understanding could potentially provide a route to improve upon recent studies where results may vary considerably between individuals [7], as it illuminates the variability in recruitment that could occur with subtly changing electrode positions. This study therefore supports the design and development of new high-density electrodes to achieve selective activation, which may improve the effects of human SCS trials. Finally, this study highlights the potential use of epidural SCS to target autonomic systems generally [70] by adding a physiological basis for stimulating these pathways. Future studies will include direct measures of LUT function in response to stimulation, as well as computational modeling to investigate the propagation of stimulation current in different tissues and identify effective stimulation parameters. Further, computational models would allow systematic investigations of the effects of changing electrode size and geometries on recruitment and how different electrodes might be designed to selectively stimulate different dorsal roots or rootlets.

## Data Availability

The data that support the findings of this study are openly available at the following URL/DOI: https://doi.org/10.26275/hbuu-caud. This dataset also contains data from other experiments; animals 1-6 described in this study are referred to in the dataset in order as subjects 54, 60, 64, 63, 68, 69, and 78. The data that support the findings of this study are openly available at the following URL/DOI: https://doi.org/10.26275/hbuu-caud [71].
